# CRISPR–Cas systems against carbapenem resistance: from proof-of-concept to clinical translation

**DOI:** 10.3389/fmicb.2025.1725247

**Published:** 2025-12-19

**Authors:** Pandora Jim Tsolakidou

**Affiliations:** 1 General Hospital of Volos, Volos, Greece; 2 Diethnes Panepistemio tes Ellados, Thessaloniki, Greece

**Keywords:** antimicrobial resistance, bacteriophage delivery, carbapenem resistance, Cas13a/Cas13d RNA targeting, Cas9 plasmid curing, conjugative CRISPR vectors, CRISPR–Cas systems, horizontal gene transfer

## Abstract

Carbapenem-resistant *Enterobacterales* (CRE) pose a major global threat, driven by plasmid-borne carbapenemase genes such as *bla*_KPC_, *bla*_NDM_ and *bla*_OXA-48_. CRISPR–Cas systems offer programmable strategies to selectively eliminate these resistance determinants. This mini-review summarizes recent advances in Cas9-based plasmid curing, RNA-targeting approaches such as Cas13a and Cas13d, and DNA-targeting Cas3-enhanced bacteriophage therapeutics that have entered early clinical evaluation. Particular attention is given to conjugative CRISPR–Cas9 plasmid systems, which enable targeted plasmid eradication without laboratory transformation and broaden the delivery toolbox beyond phage vectors. We further discuss major translational challenges, including delivery efficiency, phage host-range constraints, ecological risks of horizontal CRISPR dissemination, and off-target effects. Finally, we highlight emerging delivery platforms—outer membrane vesicles, lipid and polymeric nanoparticles, conjugative plasmids with containment circuits, and engineered live biotherapeutics—that may complement or overcome current limitations. Collectively, these developments illustrate the potential of CRISPR-based antimicrobials to augment traditional therapies through precise gene-level suppression of carbapenem resistance.

## 1 Introduction

Carbapenem-resistant *Enterobacterales* (CRE) have emerged as a major public-health threat and are listed by the World Health Organization as *critical priority pathogens* (World Health Organization, 2023). These organisms cause severe infections with limited therapeutic options and are associated with mortality rates exceeding 40% in bloodstream and intensive-care infections. Resistance is largely mediated by horizontally transferable carbapenemase genes (*bla*_KPC_, *bla*_NDM_, *bla*_OXA-48_, and *bla*_VIM_) which are often carried on highly mobile plasmids (Meletis, 2016). The global dissemination of these plasmids has outpaced the discovery of new β-lactam agents and renders traditional antibiotic development increasingly ineffective.

CRISPR–Cas systems, originally identified as adaptive immune mechanisms in bacteria and archaea, have recently been repurposed as programmable antimicrobials capable of selectively targeting resistance determinants. Unlike conventional antibiotics, CRISPR-based approaches act with nucleotide-level precision, allowing the elimination or silencing of specific genes that confer resistance (Rodrigues et al., 2019). Various Cas nucleases—Cas9 and Cas13a—have demonstrated the ability to disrupt or destroy carbapenemase genes and their plasmid vectors in experimental models (Hillary and Ceasar, 2023). Moreover, early clinical trials using CRISPR-enhanced bacteriophages in urinary and intestinal infections suggest that targeted nucleic-acid–based antimicrobial strategies may be feasible in humans (Kim et al., 2024; SNIPR Biome, 2023).

This mini-review summarizes current advances in the use of CRISPR–Cas systems against carbapenem-resistant pathogens, focusing on mechanistic diversity, preclinical findings, and translational readiness for clinical implementation.

### 1.1 Literature search

This review was based on peer-reviewed publications indexed in PubMed and Scopus (2015–2025) using the keywords *CRISPR*, *Cas13a*, *Cas9* and *carbapenem resistance*. Priority was given to original experimental studies, translational models, and clinical trials.

## 2 CRISPR–Cas mechanisms relevant to antimicrobial design

CRISPR–Cas systems constitute adaptive immune pathways that enable bacteria to recognize and cleave foreign genetic elements such as plasmids and phages. These systems are broadly divided into two classes: Class 1, which employ multi-protein effector complexes (e.g., Cas3) and Class 2, which rely on a single multidomain nuclease (e.g., Cas9, Cas12, Cas13). Their programmable nature—defined by a short CRISPR RNA (crRNA) that guides the effector to a complementary nucleic-acid sequence—forms the basis for their repurposing as precision antimicrobials (Lone et al., 2018; Shi and Wu, 2024; Javed et al., 2023; Nittayasut et al., 2024).

### 2.1 Cas9: sequence-specific DNA cleavage

The Cas9 nuclease (Type II) recognizes target DNA adjacent to a protospacer-adjacent motif (PAM) and introduces double-strand breaks through its RuvC and HNH domains. When guided to a plasmid-borne resistance gene such as *bla*_KPC-2_, *bla*_NDM-1_ or *bla*_OXA-48_, Cas9 can disrupt gene function or trigger plasmid loss. Its major strength lies in its simplicity and high cleavage efficiency (Citorik et al., 2014; Yosef et al., 2015; Hsu et al., 2013); however, potential off-target cleavage and limited *in vivo* delivery remain obstacles to therapeutic use. Cas9 is best suited for *ex vivo* plasmid curing or ex vivo plasmid curing, or for engineered probiotic applications rather than direct in-patient therapy.

### 2.2 Cas13a: RNA-guided RNA targeting

Cas13a (Type VI) is unique among CRISPR systems in that it targets RNA rather than DNA (Kiga et al., 2020). Upon binding its target transcript—such as an mRNA encoding a carbapenemase enzyme such as KPC-2—Cas13a becomes activated and nonspecifically degrades both the target and nearby RNAs, leading to bacterial cell death. Because it does not introduce double-stranded DNA breaks, Cas13a minimizes genomic integration risks and is suitable for delivery via bacteriophage capsids or nanoparticles. These features make it a highly promising candidate for clinical antimicrobial applications, capable of selectively eliminating carbapenemase-expressing bacteria while sparing nonresistant strains.

## 3 Historical context of CRISPR–Cas antimicrobials

The concept of CRISPR–Cas as a programmable antibacterial strategy was first demonstrated by Citorik et al. (2014), who used phagemid-delivered Cas9 to selectively kill *Escherichia coli* carrying antibiotic-resistance genes. Yosef et al. (2015) extended this approach using temperate phages to deliver CRISPR arrays that eliminated plasmid-encoded determinants and immunized bacterial populations against reacquisition. Subsequent refinements employed conjugative plasmid pLCasCureT to cure resistance plasmids in *E. coli* (Yen et al., 2024), while introduced RNA-targeting Cas13a constructs that enabled highly specific bacterial killing. Foundational mechanistic insights from on Cas9 PAM recognition further guided the rational design of biosafe, sequence-specific antimicrobials. Together, these pioneering investigations laid the groundwork for the carbapenem-focused studies described below.

### 3.1 Cas13-based programmable killing

Kiga et al. (2020) pioneered the use of Cas13a as an antimicrobial tool against carbapenem-resistant *Escherichia coli*. By packaging Cas13a with customized crRNAs into bacteriophage capsids (M13 or Φ80/PICI), the authors achieved sequence-specific killing of strains harboring *bla*_IMP-1_, *bla*_*NDM-1*,_ *bla*_*KPC-2*,_ *bla_VIM-2_*, and *bla_OXA-48_*. In a *Galleria mellonella* infection model, treatment with Cas13a phage targeting *bla*_IMP-1_ significantly improved larval survival. Because Cas13a acts on RNA, it eradicated cells expressing the resistance gene regardless of whether it was chromosomal or plasmid-borne. Hart et al. (2025) demonstrated that CRISPR–Cas13d enables highly precise RNA targeting with minimal off-target activity. Their work expands CRISPR applications beyond DNA editing, offering a programmable tool for post-transcriptional regulation. This level of specificity strengthens the potential of RNA-targeted antimicrobials and complements plasmid-curing strategies that act at the DNA level.

### 3.2 Cas9-mediated plasmid and gene curing

Hao et al. (2020) used a Cas9–sgRNA plasmid (pCasCure) delivered by electroporation to *Serratia marcescens*, successfully restoring carbapenem susceptibility this study demonstrated that Cas9 can eliminate carbapenemase genes (*bla_KPC_*, *bla_NDM_*, *bla_OXA-48_*) or their associated plasmids in multiple *Enterobacterales* species. After transformation with inducible Cas9–sgRNA constructs, resistant clinical isolates of *Klebsiella pneumoniae*, *E. coli*, *Enterobacter* spp., and *Serratia marcescens* lost the targeted genes and exhibited an eight-fold reduction in carbapenem minimum inhibitory concentrations. Although delivery required laboratory transformation, the study confirmed that precise DNA cleavage could restore carbapenem susceptibility in clinical strains.

A recent study by Yen et al. (2024) introduced a Cas9-based conjugative vector designed to cure common plasmids in Gram-negative bacteria. The system leverages conjugation to deliver a programmable CRISPR–Cas9 module directly into recipient cells, enabling targeted plasmid cleavage without the need for electroporation or other non-physiological delivery methods. Importantly, the conjugative vector achieved efficient plasmid elimination across multiple hosts, demonstrating broad applicability. This work highlights the clinical potential of conjugation-mediated Cas9 delivery, addressing a major limitation of earlier approaches that relied on laboratory-only transformation techniques. However, as with all conjugative systems, host range and transfer efficiency remain important considerations for *in vivo* translation.

### 3.3 Functional genomics and plasmid-fitness mapping

Calvo-Villamañán et al. (2025) employed CRISPR interference (CRISPRi) with dCas9 to perform plasmid-wide knockdowns on the *bla*_OXA-48_ plasmid across clinical *Enterobacterales* isolates. The work identified *bla_OXA-48_* itself and plasmid-stability modules as key fitness burdens, providing molecular targets for future multiplexed CRISPR antimicrobials.

A concise summary of representative studies is presented in Table 1.

**Table 1:** Representative studies using CRISPR–Cas systems against carbapenem resistance.

Target gene(s)	Study (year)	Organism(s)	Cas system	Delivery method	Main outcome	Limitations
*bla*_NDM_	Citorik et al. (2014)	*E. coli*	Cas9	Transduction bacteriophage particles (ΦRGN)	Strain specific reduction	Not clinically applicable.
*bla*_NDM-1_	Yosef et al. (2015)	*E. coli*	Cas9	Transduction temperate phage	Failure to transform the bacteria cells that contained the *bla*_NDM-1._	Transformation failure; limited efficiency with *bla*_NDM-1_.
*bla*_NDM_	Hao et al. (2020)	*Seratttia marcescens*	Cas9	Electroporation single guide RNA (sgRNA) (pCasCure)	Restored carbapenem susceptibility	Electroporation-only delivery; no clinical translation
*bla*_KPC_	Hao et al. (2020)	*Enterobacter*	Cas9	Electroporation (sgRNA)(pCasCure)	Restored carbapenem susceptibility	Electroporation-only delivery; limited applicability
*bla_OXA-48_*	Hao et al. (2020)	*K. pneumoniae isolate 49,210*	Cas9	Electroporation (sgRNA) (pCasCure)	Failure	
*bla*_KPC_	Zhu et al. (2021)	*Pseudomonas aeruginosa*	Cas9	N/D	Restored carbapenem susceptibility	Undefined delivery; no translational validation.
*bla_IMP-1_, bla_OXA-48,_ bla_VIM-2_, bla_NDM-1_, bla_KPC-2_*	Kiga et al. (2020)	*E. coli*	Cas13a	Transduction (Cas13a encapsulated with bacteriophage capsid)	Survival of infected *Galleria mellonella*	Host range of the phage capsids
*bla*_KPC-31_	Ruegsegger et al. (2022)	*Citrobacter Freundii*	Cas9	Electroporation pMB1 [vector-based plasmid pCasCure-Rif]	Restored susceptibility to ceftazidime-avibactam	Electroporation-only delivery; limited applicability
*bla*_KPC-2_ *bla*_NDM-5_	He et al. (2022)	*E. coli*	Cas9	Conjugation Tn: IS26-CRISPR/Cas9	Restored carbapenem susceptibility	Random IS26–CRISPR-Cas9 integration. Difficult to control
*bla*_KPC-2_	Tao et al. (2023)	*E. coli*	Cas9	Transformation [recombinant plasmid pCas9-sgRNA(*bla*_KPC-2_)]	Restored their susceptibility to imipenem	Not clinically applicable.
*bla*_NDM-5_	Li et al. (2022)	*E. coli*	Cas9 (sgRNA)	Transformation or conjugation delivery method	Restored their susceptibility to meropenem	Conjugation efficiency variable; potential plasmid spread.

### 3.4 Translational implications for carbapenem resistance

These early human trials directly tested several uncertainties that had persisted since the preclinical phase. In animal and *in vitro* studies, questions remained about whether CRISPR elements could persist or propagate within commensal microbiota, whether host immunity would neutralize engineered phages, and whether phage replication might trigger systemic inflammation (Hart et al., 2025). The LBP-EC01 and SNIPR001 studies began to resolve these issues: both demonstrated that CRISPR-armed phages were transient, largely confined to target bacterial populations, and did not elicit strong inflammatory or adaptive immune responses (Kim et al., 2024; SNIPR Biome, 2023).

This evidence not only supports the biosafety of CRISPR delivery but also validates the phage capsid as a scalable and immunologically tolerated vector for subsequent carbapenem-targeted constructs. By closing this feedback loop between bench and bedside, these trials shift CRISPR-based therapy from theoretical feasibility to a regulated, monitorable intervention. The challenge now lies less in if delivery works and more in how precisely it can be adapted to target carbapenemase-producing *Enterobacterales.*

For combating carbapenem resistance specifically, Cas13a-based RNA-targeting systems could logically follow this model. Phage-encapsidated Cas13a constructs capable of degrading *bla_NDM-1_* or *bla_KPC-2_* transcripts may offer sequence-specific killing with minimal risk of horizontal gene transfer. Compact RNA-targeting variants such as Cas13d (Hart et al., 2025) are also emerging as safer payloads for human application due to their smaller size and reduced collateral activity.

### 3.5 Alternative delivery methods

Beyond phage-based vectors, several alternative delivery technologies are emerging for CRISPR antimicrobial applications and may overcome some of the intrinsic limitations of bacteriophages. Nanoparticle-based platforms—including lipid nanoparticles, polymeric nanocarriers, and charge-modified inorganic particles—can encapsulate CRISPR–Cas ribonucleoproteins or guide RNAs and deliver them directly to bacterial cells (Gupta et al., 2021; Rui et al., 2020; Chowdhry et al., 2023). These systems offer advantages such as protection of the CRISPR payload from degradation, reduced dependence on species-specific receptors, and potential for targeted surface functionalization. However, nanoparticle delivery faces significant challenges, including limited penetration in biofilms and rapid clearance by host immune mechanisms. Additional non-phage strategies include outer membrane vesicles engineered to package CRISPR cargo, conjugative plasmid systems equipped with containment circuits to prevent uncontrolled horizontal transfer, and live biotherapeutics designed to transiently release CRISPR constructs within the gut (Rahmati et al., 2025). Although these approaches remain in early development, integrating them into the translational landscape provides a more realistic and comprehensive view of future CRISPR delivery pathways beyond bacteriophage-mediated systems (see Table 2).

**Table 2:** Delivery methods and limitations.

Target	Study	Cas9 delivery format	Delivery method	Advantages	Limitations
*bla* genes	Hao et al. (2020)	single guide RNA (sgRNA) (pCasCure)	Electroporation	High intracellular delivery efficiency; easy lab implementation	Severe cell viability reduction; not applicable *in vivo*; used only for proof-of-concept antimicrobial studies
*mec*A gene in *Staphylococcus aureus*	Kang et al. (2017)	Polymer-derivatized Cas9 + sgRNA (Cr-Nanocomplex)	N/D	Improved bacterial uptake; efficient genome editing; better loading (less free carrier) than non-covalent systems; higher efficiency vs. native Cas9 or lipid-based carriers	In vitro study
*cyclin-dependent kinase 5 gene*	Tu et al. (2020)	Weak acidity-responsive nanoparticles co-loaded with CRISPR/Cas9 and paclitaxel (PTX)	N/D	effective tumor growth inhibition	Unknown efficacy for bacteria
*pap*G gene	Gupta et al. (2021)	nanocomplex CRISPR-dots	Carbon quantum dots (CQDs)	UPEC had significantly reduced adherence ability and biofilm forming ability increased survival of *Caenorhabditis elegans* (*C. elegans*) worms	In vitro study
Tumor cells	Wang et al. (2025)	dCas9-ClyA protein was enveloped in OMVs	Outer membrane–derived vesicles (bacterial EVs/OMVs)		

### 3.6 Limitations of current CRISPR delivery strategies

Although CRISPR–Cas antimicrobials have shown promising proof-of-concept activity, several fundamental limitations restrict their current translational potential. The most significant constraints relate to delivery efficiency. Bacteriophage-based vectors, despite being the most advanced platforms, suffer from an inherently narrow host range, dependence on specific surface receptors, and variable stability within infected tissues. Host immune responses can neutralize incoming phage particles, while phage replication dynamics remain unpredictable *in vivo*. These limitations restrict the reliable targeting of diverse carbapenemase-producing *Enterobacterales* populations (Guo et al., 2024). Beyond phages, alternative platforms such as nanoparticles, outer membrane vesicles, and conjugative plasmids face their own challenges. Nanoparticle formulations often struggle with limited biofilm penetration, rapid clearance, and potential cytotoxicity, while their surface functionalization for bacterial targeting is still technically demanding. Conjugative plasmid systems, although effective in controlled settings, rely on successful donor–recipient contact, are influenced by competing plasmids in the microbial community, and carry the theoretical risk of horizontal transfer beyond the intended host range. Engineered live biotherapeutics also present uncertainties in colonization stability and biosafety.

Finally, all CRISPR antimicrobials must contend with bacterial escape mechanisms (Mayorga-Ramos et al., 2023). Mutations within protospacer or PAM regions, acquisition of anti-CRISPR proteins (Pinilla-Redondo et al., 2020), and structural rearrangements of resistance plasmids can impair nuclease targeting. Biofilm-associated phenotypes and heterogeneous metabolic states further reduce the uniformity of CRISPR-mediated killing. Collectively, these limitations highlight the need for continued optimization of both CRISPR design and delivery modalities to ensure robust and predictable therapeutic performance. Addressing off-target activity remains a critical safety requirement for CRISPR/Cas9-based antimicrobials (Guo et al., 2023). Unintended cleavage at partially homologous genomic sites can trigger genotoxic lesions, stress-induced mutagenesis, or unpredicted transcriptional changes in bacterial populations, particularly under infection-associated physiological states. High-fidelity Cas9 variants, rational gRNA design, and regulated nuclease expression minimize—but do not eliminate—these risks. Consequently, off-target assessment using orthogonal verification methods and stringent biosafety thresholds is essential before Cas9-based constructs can advance toward human therapeutic application.

## 4 Future perspectives and ethical considerations

### 4.1 Delivery innovation and therapeutic optimization

While delivery systems such as bacteriophages currently dominate translational efforts, several complementary strategies are emerging to overcome the inherent constraints of phage-based vectors. Compact CRISPR variants and RNA-guided effectors with reduced payload size may broaden compatibility across delivery platforms, including nanoparticles, outer membrane vesicles, and engineered live biotherapeutics (Rahmati et al., 2025). These approaches aim to address limitations such as narrow host range, receptor dependence, and variable phage stability *in vivo*.

A parallel challenge involves fine-tuning the in vivo activity of CRISPR systems. Heterogeneous bacterial physiology, stress responses, and biofilm architecture can reduce the efficiency of nuclease targeting. Controlled-expression circuits, self-limiting designs, and multiplexed guide strategies may enhance precision while reducing the likelihood of bacterial escape. Integrating delivery innovations with real-time CRISPR-based diagnostics could ultimately enable a more adaptive and targeted therapeutic framework.

### 4.2 Ethical and ecological dimensions

Unlike chemical antibiotics, CRISPR-based agents act on the genetic fabric of microbial populations, which means their ecological footprint may extend beyond the immediate infection site. Microbial communities evolve under constant selective pressure; any intervention that introduces a new genetic weapon can, over time, select for phage resistance, anti-CRISPR proteins, or CRISPR-tolerant variants (Hillary and Ceasar, 2023). Such adaptive countermeasures could reshape resistance reservoirs rather than eliminate them.

Anticipating these evolutionary responses is therefore as critical as ensuring biosafety. Modeling population-level dynamics and incorporating “evolutionary containment”—through self-limiting phages, inducible circuits, or temporally restricted expression of CRISPR payloads—may prevent long-term ecological distortion. These considerations place CRISPR antimicrobials within the broader framework of evolutionary stewardship, where success is measured not only by immediate pathogen eradication but by maintaining the stability of microbial ecosystems.

From a clinical-ethical perspective, informed consent and risk communication will require special attention, as patients may perceive CRISPR treatment as “gene therapy,” even when directed exclusively at microbes. Transparency about off-target effects, phage persistence, and potential microbiome alterations will be essential to maintain public trust.

A parallel discussion is unfolding at the regulatory level. Agencies differ in how they classify CRISPR-enhanced biologics: the U.S. FDA currently treats bacteriophage products delivering CRISPR payloads as live biotherapeutic agents or biologic antibacterials, evaluated under frameworks similar to phage therapy; the European Medicines Agency (EMA), meanwhile, has considered such constructs under the gene therapy umbrella when the CRISPR component exerts heritable genetic modification within microbial populations. This divergence matters for trial design, biosafety thresholds, and long-term surveillance obligations. Clarifying whether CRISPR antimicrobials fall under the rules for conventional biologics or gene-therapy medicinal products will shape how—and how quickly—they move from experimental use to regulated therapy. Among regulatory authorities, the EMA maintains a stricter classification framework than the FDA, treating CRISPR-enhanced bacteriophage products as gene therapy medicinal products when heritable genetic modification is involved, whereas the FDA applies a more functional, biologics-based assessment (European Medicines Agency Amsterdam, The Netherlands, 2018; U.S. Department of Health and Human Services, Food and Drug Administration, Center For Biologics Evaluation and Research Silver Spring, MD, 2022, 2023; Science and regulation of bacteriophage therapy, Silver Spring, MD, 2023).

### 4.3 Outlook

CRISPR antimicrobials are unlikely to replace conventional antibiotics, yet they are redefining what “therapy” means in microbiology—from chemical inhibition to genetic correction. Among the current systems, Cas13a stands closest to clinical readiness: its RNA-guided activity avoids double-strand DNA breaks and has been shown to mediate sequence-specific killing of carbapenemase-producing *E. coli in vivo* (Shi and Wu, 2024; Li et al., 2022). Cas13a payloads are small enough for packaging into bacteriophage capsids or nanoparticles, and their biosafety has indirect human validation through early CRISPR-phage trials, notably the Cas3-armed LBP-EC01 program (Kim et al., 2024).

## 5 Conclusion

Carbapenem resistance represents one of the most urgent challenges of modern infectious disease medicine. Traditional antibiotics and stewardship efforts, though essential, are increasingly insufficient against mobile carbapenemase genes such as *bla*_KPC_, *bla*_NDM_, and *bla_OXA-48_*. The rapid evolution and horizontal transfer of these determinants demand new therapeutic concepts capable of acting with genetic precision rather than chemical broadness.

CRISPR–Cas systems fulfill this need by enabling selective removal or silencing of resistance genes. Over the past decade, advances in Cas9 system have demonstrated that specific targeting of carbapenemase genes can either restore susceptibility or directly eliminate resistant bacterial populations. Phage and conjugation-based delivery models have achieved proof-of-concept success *in vitro* and *in vivo*, and early-phase human trials using CRISPR-enhanced phages have confirmed safety and feasibility.

Among available tools, Cas13a holds particular promise for clinical application due to its RNA-guided activity and absence of genomic cleavage, while Cas3 and Cas9 platforms continue to offer powerful strategies for plasmid eradication and microbiome engineering. The convergence of these technologies with rapid CRISPR-based diagnostics may enable an integrated framework of “detect and destroy,” capable of targeting resistance genes before they disseminate.

The path to clinical use will require careful regulation, biosafety validation, and ethical transparency, but the progress achieved thus far signals a paradigm shift. CRISPR-based antimicrobials may not replace antibiotics, yet they have the potential to redefine therapy—transforming infection control from empiric treatment into precise molecular intervention. In the fight against carbapenem resistance, CRISPR represents not merely a new tool, but a new philosophy: the deliberate editing of resistance itself.
